# *Drosophila* Atlastin regulates synaptic vesicle mobilization independent of bone morphogenetic protein signaling

**DOI:** 10.1186/s40659-023-00462-1

**Published:** 2023-09-14

**Authors:** Francisca Bertin, Jorge Jara-Wilde, Benedikt Auer, Andrés Köhler-Solís, Carolina González-Silva, Ulrich Thomas, Jimena Sierralta

**Affiliations:** 1 Biomedical Neuroscience Institute (BNI), Santiago, Chile; 2 Department of Neuroscience, Faculty of Medicine, Universidad de Chile, Santiago, Chile; 3 SCIAN-Lab, Biomedical Neuroscience Institute (BNI), Santiago, Chile; 4 Department of Computational Sciences, Faculty of Physical and Mathematical Sciences, Universidad de Chile, Santiago, Chile; 5 Laboratory of Neuronal and Synaptic Signals, Department of Cellular Neuroscience, Leibniz Institute for Neurobiology, Magdeburg, Germany; 6 Functional Genetics of the Synapse, Department of Cellular Neuroscience, Leibniz Institute for Neurobiology, 39118 Magdeburg, Germany

**Keywords:** Atlastin, Synaptic vesicle, Vesicle mobilization, Endosome, *Drosophila*, Presynaptic terminal

## Abstract

**Background:**

The endoplasmic reticulum (ER) contacts endosomes in all parts of a motor neuron, including the axon and presynaptic terminal, to move structural proteins, proteins that send signals, and lipids over long distances. Atlastin (Atl), a large GTPase, is required for membrane fusion and the structural dynamics of the ER tubules. Atl mutations are the second most common cause of Hereditary Spastic Paraplegia (HSP), which causes spasticity in both sexes’ lower extremities. Through an unknown mechanism, Atl mutations stimulate the BMP (bone morphogenetic protein) pathway in vertebrates and Drosophila. Synaptic defects are caused by *atl* mutations, which affect the abundance and distribution of synaptic vesicles (SV) in the bouton. We hypothesize that BMP signaling, does not cause Atl-dependent SV abnormalities in Drosophila.

**Results:**

We show that *atl* knockdown in motor neurons (Atl-KD) increases synaptic and satellite boutons in the same way that constitutively activating the BMP-receptor Tkv (thick veins) (Tkv-CA) increases the bouton number. The SV proteins Cysteine string protein (CSP) and glutamate vesicular transporter are reduced in Atl-KD and Tkv-CA larvae. Reducing the activity of the BMP receptor Wishful thinking (*wit*) can rescue both phenotypes. Unlike Tkv-CA larvae, Atl-KD larvae display altered activity-dependent distributions of CSP staining. Furthermore, Atl-KD larvae display an increased FM 1–43 unload than Control and Tkv-CA larvae. As decreasing *wit* function does not reduce the phenotype, our hypothesis that BMP signaling is not involved is supported. We also found that Rab11/CSP colocalization increased in Atl-KD larvae, which supports the concept that late recycling endosomes regulate SV movements.

**Conclusions:**

Our findings reveal that Atl modulates neurotransmitter release in motor neurons via SV distribution independently of BMP signaling, which could explain the observed SV accumulation and synaptic dysfunction. Our data suggest that Atl is involved in membrane traffic as well as formation and/or recycling of the late endosome.

**Supplementary Information:**

The online version contains supplementary material available at 10.1186/s40659-023-00462-1.

## Background

Motor neurons mediate communication between the motor centers in the brain and the musculoskeletal system, which is necessary for every voluntary movement in an organism [1]. Membrane trafficking processes play a fundamental role in the development and function of motor neurons, which are highly polarized, excitable secretory cells. Among the proteins necessary for membrane traffic, Rab GTPase proteins [2, 3] are essential for vesicle traffic as well as for vesicle and endosome biogenesis through their role in membrane fusion [4], which is in turn, essential for cellular maintenance, synaptic communication, and signaling pathways [5–7]. The endoplasmic reticulum (ER) displays an extended presence in neurons, reaching from the nuclear envelope to the plasma membrane and into the axonal presynaptic terminal. The ER also has close communication with multiple organelles such as endosomes and mitochondria; these characteristics support its role as a long-distance intracellular connector and regulator of signaling [8–10] ER-organizing proteins like Atlastin (Atl) are essential for ER-mediated functions because they enable structural flexibility through the regulation of their contact network [11]. Atl is a large GTPase that belongs to the dynamin superfamily. It is found in the ER, Golgi apparatus, and endosomes [12–14], where it is responsible for homotypic membrane fusion. This is what gives the axonal tubular ER network its characteristic orthogonal shape [13, 15]. In mammals, Atl is expressed by 3 different genes of ample expression (Atl-1 to 3), with Atl-1 expressed mainly in the nervous system, where it regulates the morphogenesis and dynamics of the Golgi apparatus and the ER structural changes. In endosomes, it has been suggested that it exerts a regulatory role in membrane traffic [16].

In *Drosophila*, only one *atlastin* (*atl*) gene has been described with a role in trans-synaptic signaling modulation, specifically a downregulation of BMP (Bone Morphogenic Protein) signaling [14, 17]. BMP signaling at the neuromuscular junction (NMJ) is a homeostatic signal that involves retrograde signaling by the muscle-derived ligand Gbb (Glass Bottom Boat) onto serine/threonine kinase receptors Wit (Wishful Thinking), Tkv (Thick Veins), and Sax (Saxophone), in the motor neuron. [18–20]. The activation of the receptors promotes the dimerization and phosphorylation of the signaling complex (Wit/Tkv or Wit/Sax), which is endocytosed and trafficked retrogradely in signaling endosomes to the soma of the neuron, where it phosphorylates the cytosolic protein MAD (Mothers Against Decapentaplegic, homologous to mammalian SMAD). pMAD translocate to the nucleus, promoting the transcription of genes such as *trio* [21–23]. In the terminal, a local increase of phosphorylated MAD in the synaptic bouton is also detected, which is involved in synaptic development independent of the nuclear signal [23, 24]. BMP signaling involves modifications in the actin cytoskeleton and microtubules, and phenotypically, its hyperactivation promotes the formation of satellite boutons, corresponding to small ectopic boutons that emerge from pre-existing synaptic boutons or the main terminal branch of the NMJ [25, 26]. In *Drosophila*, an interactome study related Atl with Rab4, which is involved in the rapid recycling of endocytosed components and mediates the redistribution of cargo from early endosomes to the plasma membrane, either towards Rab11-positive late recycling endosomes or to Rab7-positive late degradation compartments [6, 27].

In *Drosophila*, *atl* null mutations lead to progressive motor defects, increased satellite bouton numbers, and ER structural abnormalities in the larval NMJs. Elevated nuclear pMAD in the motor neuron is a sign of increased BMP signaling that is concurrent with these phenotypes [17, 28, 29]. Atl-KD larvae exhibit an accumulation of synaptic vesicle (SV) and lysosome markers in distal axons, with a reduced SV number in the area surrounding the active zone (a region that has been associated with the recycling vesicle pool) and defects in the recovery of synaptic function after a tetanic stimulus, which is compatible with the morphological defect [28]. Although mutations in repressor proteins of BMP signaling (implying an increase in this signaling) also generate a reduction in SV number [26, 30, 31], the number and size of the vesicles are disrupted within the active zone, a phenotype not observed in Atl knockdown (Atl-KD) larvae [28]. Additionally, larvae with increased BMP signaling in motor neurons, do not display axonal accumulation of SVs as was detected in Atl-KD larvae [26, 30, 31]. These phenotype differences between increased BMP signaling induced by Atl or by direct upregulation of the pathway suggest that Atl loss of function affects a wider set of functions besides BMP signaling, including SV intracellular trafficking.

We hypothesized that Atl knockdown, affects SV intracellular trafficking, independently of the increase in BMP signaling. Our results support this hypothesis and suggest that the loss of function of Atlastin produces the accumulation of SV in axons and defects in SV number surrounding the active zone through an undescribed role in the regulation of endosomal trafficking. Alterations in ER morphology and function have been associated with the development of several neurodegenerative diseases, such as Huntington's disease, Amyotrophic Lateral Sclerosis, and Hereditary Spastic Paraplegia (HSP) [32–35]. Autosomal *atl* dominant mutations represent 10% of HSP cases [36, 37], but mutations associated with ER proteins represent more than 60% of the cases, including the early onset type of the disease [33, 36, 37]. Thus, our results showing that SV distribution and neurotransmitter release defects are part of the pathological mechanism underlying the distal axonopathy present in Atl-associated HSP and that they could be ligated to defects in ER membrane trafficking contribute to the understanding of the disease and potentially to the search for new treatments for the pathology.

## Results

### Atl-KD in motor neurons increases BMP signaling

**Fig. 1:**
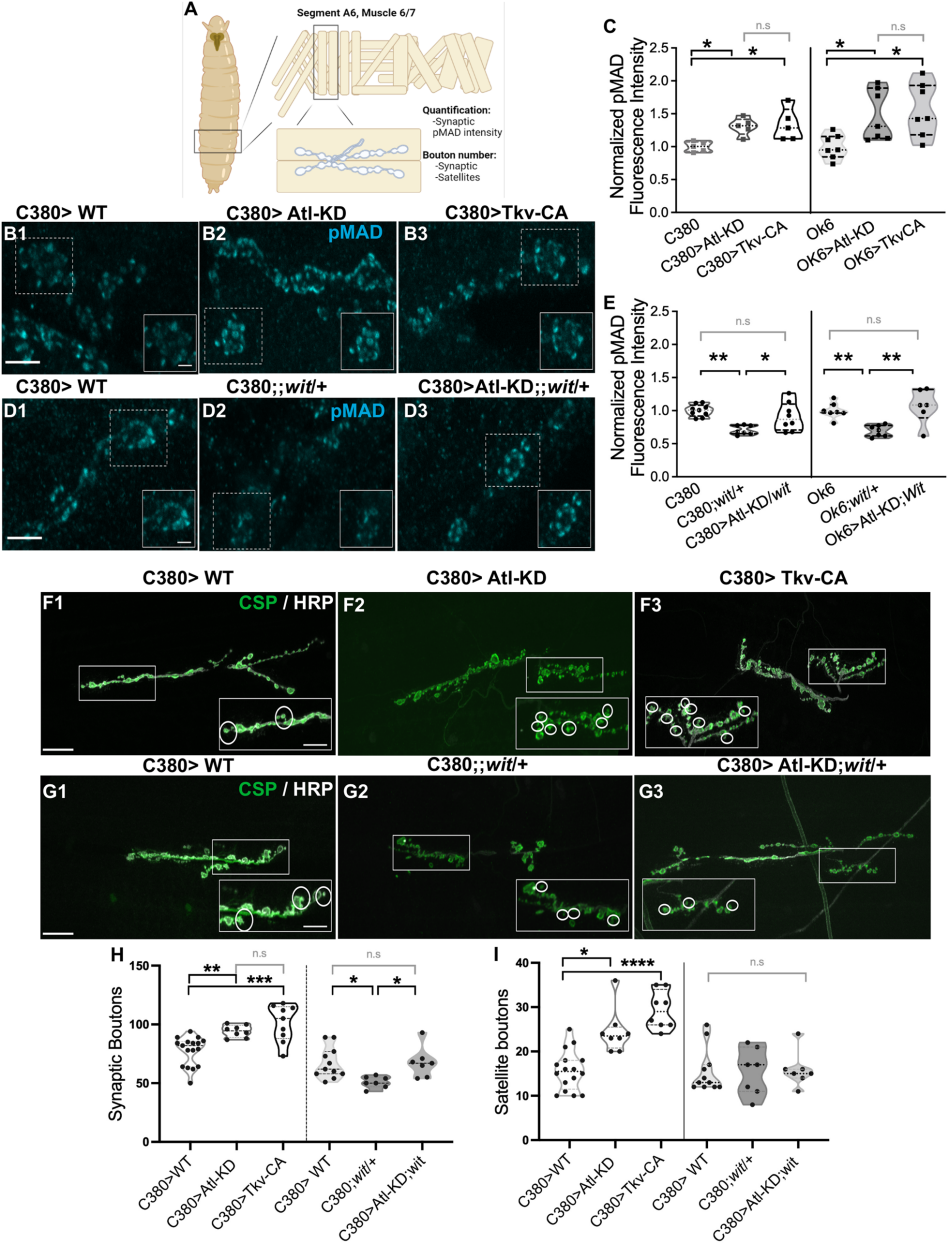
Atl-KD in motoneurons increases synaptic pMAD and morphometric parameters **A** Illustration of the *Drosophila* larvae body wall preparation, the area that is imaged in the following pictures and the synaptic boutons of the NMJ Cartoon created with BioRender.com. **B**, **C** Representative confocal microscopy images (maximum intensity Z-projection) of synaptic boutons immunostained for pMAD **B** and the quantification of its intensity in larvae expressing Atl-KD or Tkv-CA using the driver C380 **B** and **C** or OK6 **C**: pMAD intensity was normalized to the control pMAD intensity levels (Driver C380 or Ok6, respectively). D, E) Representative confocal microscopy images (maximum intensity Z-projection) of synaptic boutons immunostained for pMAD **D** and the quantification of its intensity in larvae heterozygous for the *wit* mutation alone or with Atl-KD using the drivers C380 **D** and **E** and OK6 **E**. pMAD intensity was normalized to the control pMAD intensity levels (C380 or OK6, respectively). pMAD antibody staining is in cyan color. Scale bar of large image: 5 µm, of cropped image: 2 µm. **F**–**G** Representative confocal microscopy images (maximum intensity Z-projection) of presynaptic terminals of larvae of the different genotypes labeled with CSP and HRP antibodies staining in green and gray color, respectively. In cropped images, satellite boutons are indicated by white circles. Image intensity and contrast have been increased for visualization purposes. Scale bar 20 µm and 10 µm in the magnified insert. **H**–**I** Quantification of synaptic and satellite bouton number of the genotypes in **F**–**G**. Each dot represents one larva. Kruskal-Wallis, p-value * < 0,05; ** < 0.01; *** < 0.001. n = 5-9

In *Drosophila*, *atl* null mutant larvae display an increase in neuronal nuclear pMAD accumulation as well as an increase in the number of synaptic boutons, a phenotype associated with enhanced BMP signaling [17, 28]. To confirm that the phenotype observed in motor neurons is the result of increased neuronal BMP signaling, we measured synaptic pMAD accumulation in larvae with motor neuron *atl* knockdown *(Gal4 xUAS-Atl-RNAi* (Atl-KD), Fig. 1A–E). In our previous work, we used two different RNAi constructs, one double strand (dsRNA) and one small interferent of the TRiP family, that gave us essentially the same results [28]. Here we used only the dsRNA in combination with Dicer overexpression, which displays a slightly stronger phenotype than the TRiP construct. Importantly, we focused our analyses on the synaptic boutons of the neuromuscular junction on muscles 6 and 7 of the abdominal segment A6, where the motor axons are the longest and thus particularly prone to distal axonopathy (showed in De Gregorio et al. [42]). As a positive control, we evaluated by immunostaining the pMAD accumulation in synaptic boutons of larvae with an overexpression of the constitutively active Thickveins receptor (Tkv-CA). Both *atl*-KD and Tkv-CA expressions were carried out using two different *Gal4* with motor neuron expressions: *C380-GAL4* and *OK6-GAL4*. Tkv-CA expression with either Gal4 driver induced a significant increase in synaptic pMAD (33% and 56%, respectively, Fig. 1B–C) evaluated by immunostaining. A similar increase in synaptic pMAD was observed in Atl-KD larvae (30% for the *C380-GAL4* promoter and 47% for *OK6-GAL4*, Figs. 1B–C; Additional file 1: Figure S1). Therefore, the increase in neuronal BMP signaling observed in *atl* mutants [17] can be replicated by neuronal Atl-knockdown. To confirm that synaptic pMAD accumulation in Atl-KD larvae requires the activation of BMP receptors, we measured the synaptic pMAD in Atl-KD larvae with a decreased allelic dose of the Wit receptor using a *wit* null mutant allele (Atl-KD/*wit*). While pMAD levels in heterozygous *wit/* + larvae showed a 31% reduction, heterozygosity for this mutation in Atl-KD larvae suppressed the increase of synaptic pMAD levels (Fig. 1D–E, Additional file 1: Figure S2). Thus, we confirm that neuronal Atl-KD replicates the *atl* mutant BMP activation phenotype, which requires BMP-neuronal receptors.

### Atl-KD and BMP activation in motor neurons increase synaptic and satellite bouton number

Altered synaptic function in the *Drosophila* larval NMJs is often found associated with variations in neuronal morphology, such as the occurrence of the so-called satellite boutons. Satellite boutons refer to small, immature, supernumerary boutons that emerge from a parental bouton [38]. Mature boutons characterize by their size and the presence of several active zones (labeled by anti-Bruchpilot antibody). Loss of *atl* function generates an increased number of satellite boutons along with deficiencies in synaptic function [17, 28]. An increase in satellite bouton number has been associated with BMP activation, related to changes in the organization of the actin and microtubule cytoskeleton [21, 39]. However, such increases have also been described in some endocytic mutants where BMP signaling modifications have not been reported, suggesting that these boutons could emerge as a compensatory response to endocytic failure [40]. C380-driven Atl-KD and Tkv-CA in larval NMJs show a significant and similar increase in the number of synaptic boutons (47%, Fig. 1F, H, Atl-KD, and 31%, Fig. 1F, H, Tkv-CA) compared to control animals. A similar result was obtained in the quantification of the number of satellite boutons in Atl-KD larvae (66% of the total bouton number, Fig. 1F, I) and Tkv-CA larvae (61%, Fig. 1F, I). Similar findings were made using *OK6-Gal4* instead of *C380-Gal4* (Additional file 1: Figure S1). To determine the contribution of BMP signaling to the Atl-KD dependent-NMJ structural phenotype, again we quantified mature and satellite boutons in *wit/* + larvae and in Atl-KD NMJs in a *wit*/ + background (Atl-KD/*wit*). We found that *wit/* + mutation significantly reduces the number of synaptic boutons by 24% and suppresses the increase of synaptic boutons in Atl-KD larvae (Figs. 1G–H). Regarding the number of satellites, *wit/* + mutants, and controls, they were indistinguishable. However, a decreased dose of *wit* prevented the bouton satellites' increase in Atl-KD larvae. (Fig. 1G, I). This morphometric analysis shows that the increased number of synaptic and satellite boutons is a result of an increase in BMP signal, which is caused by Atl-KD in motor neurons.

### Atl-KD increases CSP peripheral density after stimulation

**Fig. 2:**
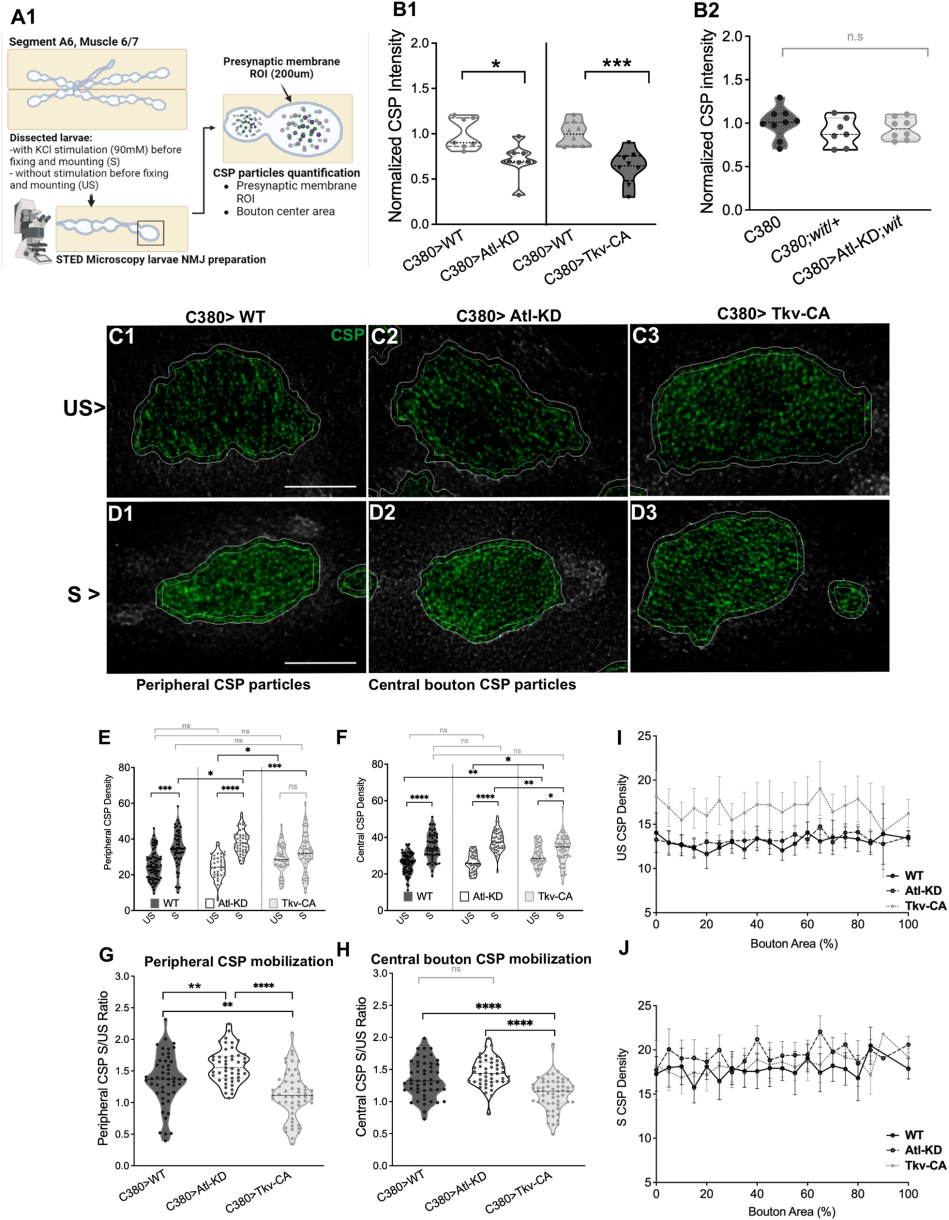
Synaptic stimulation increases peripheral CSP density in Atl-KD larvae **A** Illustration showing the protocol followed for the acquisition of NMJs STED images, with (S) or without (US) KCl Stimulation; larvae were fixed and processed for immunostaining after the protocol. Quantification of the CSP particle density was performed in the peripheral delimited area (ROI generated from the external limit of HRP staining to 200 µm inside the bouton), as well as in the center of the bouton. Cartoon created with BioRender.com. **B** Normalized CSP intensity in larval boutons of different genotypes. Each dot represents the average density of at least 10 boutons in one larva. ** = Mann-Whitney p-value < 0.01. n = 7-9 larvae. **C**–**D** Representative images of CSP staining in synaptic boutons of Atl-KD and Tkv-CA larvae. Scale bar: 2 µm. **E**–**F** CSP density in boutons of unstimulated (US) and KCl-stimulated (S) NMJs. Peripheral **E** and central **F** CSP density of Atl-KD and Tkv-CA larvae. **G**–**H** Vesicle mobilization (CSP density in S boutons)/(CSP density in US boutons) in Atl-KD and Tkv-CA larvae, in the periphery **G** and the center of the bouton **H** each data point represents one bouton from 5 different larvae. **I**–**J** CSP density distribution on the whole synaptic bouton by 10% segment bands, under non-stimulated (US) and stimulated (S) conditions, for the different genotypes. Each data point represents the average of all boutons with CSP particles in that segment band. One-way ANOVA, p-value * < 0,05; ** < 0.01; *** < 0.001; **** < 0.0001. n = 5 larvae

De Gregorio et al. [42] observed that Atl-KD in motor neurons reduced synaptic vesicles (SV) by employing electron microscopy. The same publication showed a striking axonal accumulation of the SV marker CSP, which colocalized with the lysosomal marker Lamp2. In *Drosophila* larvae overexpressing the Tkv-CA receptor, this axonal phenotype has not been described [26], but endosomal protein mutations that affect the BMP pathway have been reported to alter the size and abundance of SV, indicating that this signaling could be responsible for this trait [30, 31, 40]. To further investigate this phenotype and the effects of BMP signaling on the SV accumulation at the axon terminal, we used antibodies against the SV proteins CSP and the synaptic-vesicle glutamate transporter, dVGLUT, to quantify the SVs in Atl-KD and Tkv-CA larvae. CSP and dVGLUT markers decreased in abundance in both genotypes (Fig. 2A and Additional file 1: Fig. S3A). We measured CSP in Atl-KD in the *wit*/ + background to ascertain the role of BMP in this phenotype. Heterozygous *wit* mutation alone did not significantly alter CSP labeling; but it inhibited the Atl-KD-induced decrease of CSP staining (Fig. 2A1, Additional file 1: Figure S2). Thus, in motor neurons, Atl-KD, and BMP stimulation both decreased the buildup of synaptic vesicle markers in the bouton. Moreover, as larvae with the *wit*/ + mutation added to Atl-KD did not show the CSP staining drop, is possible to conclude that BMP signaling is associated to the SV phenotype seen in Atl-KD larvae (Fig. 2A2).

De Gregorio et al. [42] electron microscopy analysis shows differences between the SV distribution in Atl-KD and the mutants with enhanced BMP signaling. Considering this, we analyzed the SV marker distribution in unstimulated and stimulated conditions using STED (Stimulated emission depletion) microscopy. CSP distribution analysis was performed in the periphery of the bouton (first 200 nm from the limit of the bouton detected by the HRP signal), which is usually associated with the recycle pool of the SV, and in the center part of the bouton (the bouton area minus the periphery, traditionally associated with the reserve pool, [41].

In this analysis, we included only synaptic boutons between 2.5 and 12 µm^2^ (type Ib, size distribution observed). Comparing the CSP density observed in the periphery of the bouton, before and after the stimulus, control larvae displayed a significant increase after the stimulation, showing that the CSP labeling was able to detect the mobilization of SV triggered by KCl (Fig. 2C, D). In the Atl-KD group, as in control larva, a significant increase in the peripheral CSP labeling was observed upon KCl treatment (Fig. 2C–E). This increase was, however, significantly larger than the observed in the control (Fig. 2C–E). Notably, no variation between unstimulated and stimulated boutons was observed in Tkv-CA larvae (Fig. 2C–E). The same analysis now in the central area of the boutons, showed again a significant increase in the CSP density induced by potassium in control larvae (Fig. 2F) and in Atl-KD larvae, however, in Tkv-CA larvae CSP density showed only a moderate though significant increase after stimulus, which was associated with a higher density in basal conditions (prior to stimulation) compared to control (Fig. 2F). To better observe the differences, we compared the ratio of CSP density between US and S conditions in the periphery and in the center of the boutons. This parameter represents the mobilization of SV. The ratio in the periphery of the synaptic bouton in Atl-KD group was higher compared to its genetic control (Fig. 2G), meaning that the mobilization of the SV to the periphery was bigger in these boutons compared to control. In contrast, Tkv-CA larvae showed a reduced ratio (17%) compared to control in the periphery (Fig. 2G). Moreover, Tkv-CA larvae showed a reduced ratio in the center of boutons (18%) compared to control (Fig. 2H).

We performed a complementary analysis to examine potential changes in the SV distribution in the whole button region. All the synaptic boutons visible in the photos were included in this analysis. To enable a comparative analysis for boutons of various sizes, the bouton area was divided into segments of 10% of the bouton area and the density of each segment both before and after the stimulus, was quantified. CSP density of each segment was plotted from the center of the bouton (0%) to the periphery (100%, total area) (Fig. 2I–J). Before stimulation, Tkv-CA larvae had greater CSP densities than control and Atl-KD larvae. The three genotypes increased the CSP density after the stimulation. We conducted a second study, classifying the data in quartiles, to compare genotypes since the analysis by Decyl was highly varied. With the help of this methodology, we show that in all quartiles, Tkv-CA CSP densities were significantly higher than those in control and Atl-KD. Tkv-CA displayed greater CSP densities in the central and outer quartiles following the stimulus, but Atl-KD did not deviate from control in any quartile area (Additional file 1: Figure S5).

### Atl-KD in motor neurons modifies synaptic vesicle dynamics and distribution through recycling components in *Drosophila* NMJ

**Fig. 3:**
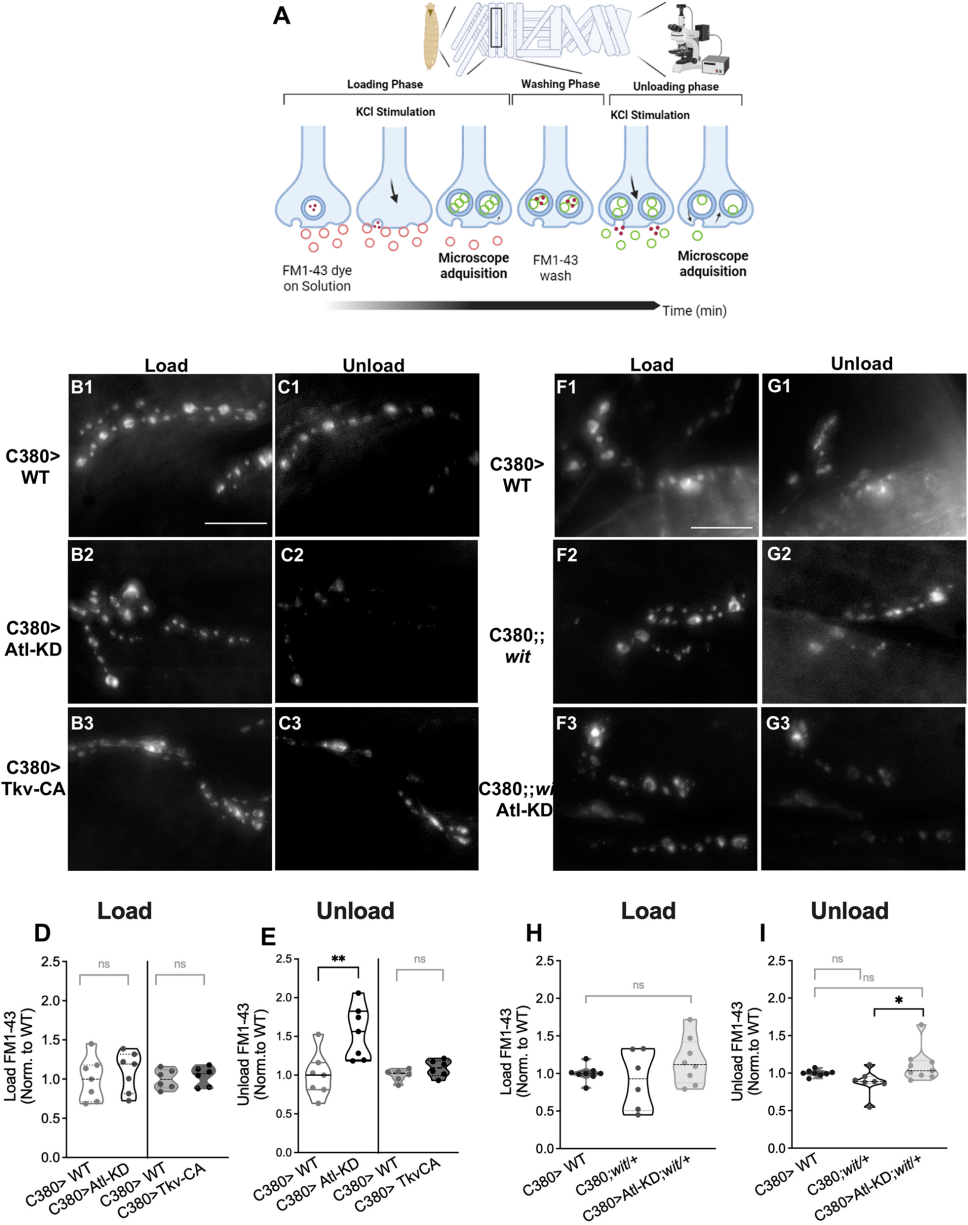
Atl-KD in motor neuron increases FM 1–43 unloading. **A** Illustration showing FM1-43 technique to evaluate SV dynamics on *Drosophila* larval NMJ, see the text for further details. Cartoon created with BioRender.com. **B**–**G** Black and white representative spinning disc images (maximum intensity Z projection) of NMJ boutons of non-fixed preparation loaded with FM1-43 dye of different genotypes. Images after load (B1-3 and F1-3) and unload (C1-3 and G1-3) protocols. Image intensity and contrast have been increased for visualization purposes. Scale bar: 20 µm. **D**, **H** quantification of the fluorescence intensity of FM 1–43 normalized to control levels after the loading protocol. **E**, **I** quantification of the fluorescence intensity of FM 1–43 normalized to control levels after the unloading protocol. Each data point represents the average of several boutons in one larva. Kruskal–Wallis, p-value * < 0,05, ** < 0.01, n = 6–9 larvae

Using the lipophilic dye FM1-43, whose discharge (unloading) of the previously endocytosed dye (loading), reflects the SV release, we investigated the dynamics of the SV (endocytosis and exocytosis) to confirm our findings. To activate the exocytosis and subsequent membrane recycling of the SVs, dissected larvae with the brain still attached were treated with 90 mM KCl for 3 min in the presence of the FM1-43 dye (see methods). During the endocytic process, the FM1-43 incorporated in the membranes will label the SV. After several washes to remove the dye from the plasma membrane, the larvae were imaged in medium containing 0.5 mM EGTA and no calcium added; this image represented the loading parameter. Then, a second stimulation was carried out to induce the exocytosis of the tagged vesicles, using the same KCl solution as before but without the dye. After the stimulation and during the image acquisition process, the larvae were maintained in buffer with EGTA and no calcium (Fig. 3A). The average fluorescence of each synaptic bouton following the second stimulation, normalized by the average load of the bouton, is subtracted from 1 to determine the unload parameter. We observed that regardless of the expression promoter employed, Atl-KD in motor neurons does not significantly alter FM 1–43 load; nonetheless, these larvae showed greater FM 1–43 unloading when compared to the control (Fig. 3D–ES1J–K). To determine whether BMP signaling was associated to this phenotype, we repeated the experiments in larvae carrying the *wit*/ + mutation. This mutation did not have effect on the dye's load or unload parameters (Fig. 3H–I). Even more, Atl-KD; *wit* larvae showed an increased unload than the *wit* larvae and the control larvae, like Atl-KD larvae, showing that the BMP signal is unrelated to the SV phenotype (Fig. 3I).

**Fig. 4:**
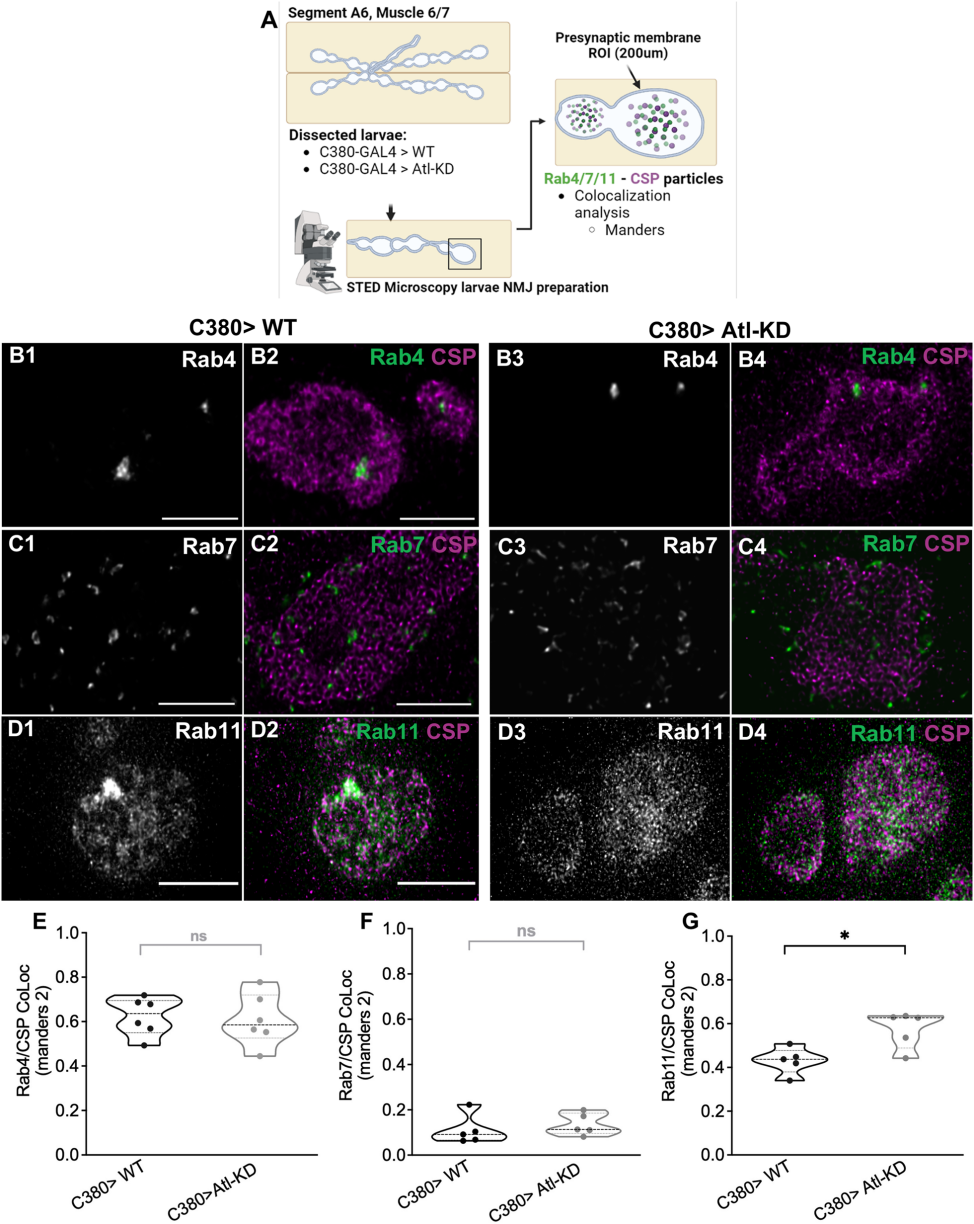
Atl-KD in motor neurons increases the colocalization of Rab11 with CSP. **A** Illustration of the protocol for acquiring STED images from WT and Atl-KD *Drosophila* NMJ, using UAS constructs Rab 4, Rab 7 and Rab 11-GFP fusion proteins overexpressed in motoneurons with C380 driver. Cartoon created with BioRender.com. B-D) STED microscopy images (maximum intensity Z projection) of control (C380) and Atl-KD NMJ boutons expressing a UAS-Rab4-GFP **B**, endogenous Rab7-GFP **C** and UAS-Rab11-GFP **D**. Scale bar: 2 µm. CSP antibody stain is represented in Magenta. Image intensity and contrast have been increased for visualization purposes. Scale bar: 2 µm. Manders 2 coefficients (GFP signal colocalized with CSP marker, divided by total GFP signal) of Rab4 **E**, Rab7 **F**, and Rab11 **G**. Each scatter dot represents one larva. Mann–Whitney, p-value * < 0,05, n = 5–6 larvae

These results, show that the changes in distribution and density of CSP in response to the stimulus, associate to functional changes in the SV release and that a mechanism independent of BMP underlies the phenotype. As we observed these defects in the mobilization of SV, we explored whether Rab-proteins involved in endocytosis and recycling displayed an abnormal distribution. For these experiments we used UAS constructs of Rab-GFP fusion proteins overexpressed using a motor neuron Gal4 promotor. To determine the colocalization of these Rab proteins with the SV, they were labeled with anti-CSP antibody and the co-distribution of the antibody labeling with GFP was quantified (Fig. 4A). Rab-4, which identifies early recycle compartments, and Rab-7, which flags the late endosome, did not show any variations in the co-distribution with CSP immunostaining [coefficients Manders 1(M1) and Manders 2(M2)] comparing Atl-KD larvae with control (Fig. 4B, C, E, F). However, Rab11, a marker for late recycling compartments, showed a significant difference in M2 coefficient (Fig. 4D, G). M1 refers to the colocalization of CSP in RAB 11 considering all CSP signals, whereas M2 represents the colocalization of the Rab11 signal on CSP labeling, normalized by the total RAB11 signal. Rab 11 is substantially less distributed and diffuse than the CSP signal, making the M1 less useful. M2 value, though, indicates that Rab11 is more associated to the SVs in the Atl-KD group than in the control group. This finding suggests that Atl-deficient vesicles accumulate in the late endosome compartment and that Atl may thus facilitate the movement of SV from the late recycling compartment to the releasable pool. Furthermore, in control boutons, Rab 11 labeling is more concentrated at a region, different from Atl-KD boutons, in which the Rab11 labeling is observed in small puncta. This type of staining could indicate vesiculation or disruption of the recycling compartment in Atl-KD.

## Discussion

Loss of Atl function has been linked to an increase in BMP signal in the *Drosophila* NMJ and in other model systems [14, 17]. Therefore, the question we set out to answer was whether the synaptic *atl* phenotype exclusively resulted from the BMP pathway's defects or alternatively if it is dependent on this pathway only partially. We show in this work that motor neuron knockdown of Atl is sufficient to significantly increase synaptic pMAD accumulation, as well as the number of synaptic boutons and satellite boutons at the NMJs, and that both phenotypes are related to increased BMP signal. Additionally, our findings demonstrate that Atl-KD larvae with reduced expression of the BMP receptor, Wit, reversed the changes in these morphometric parameters (Fig. 1), confirming that Atl’s induction of an increase in BMP signal contributes to the morphological phenotype observed in atl mutants.

Another phenotype described by De Gregorio et al. [42] was the reduction in the number of SV in the periphery of the active zone. Activation of the BMP pathway-related mutations is similarly linked to a decrease in the number of SV, but this vesicle reduction is primarily localized in the vicinity of the active zone [43, 44]. We determined that neuronal Atl-knockdown as well as the increase in BMP signaling reduce the fluorescence intensity and the number of the particles positive for the SV proteins, CSP, and VGLUT (Fig. 2A), indicating a general reduction of SVs inside the bouton and a role for Atl and BMP signaling in the correct production or recycling of SV. Interestingly, SV distribution following stimulation varied between Atl-KD larvae and TKV-CA larvae. Whereas larvae with overactivation of BMP signaling alone, display increased CSP levels in the center of the bouton and no changes in the periphery after stimulation; larvae with Atl-KD show CSP density increased in the periphery, with no significant changes in the center of the bouton post-stimulation (Fig. 2). This finding demonstrates that, despite both genotypes increasing BMP signaling, the SV abundance exhibits distinct changes in the organization of the SV after stimulation, suggesting distinct underlying processes.

The changes seen after neurotransmitter release stimulation reflect the mobilization of the SV within the bouton, in which strong and sustained synaptic stimuli increases the SV fraction of the exo-endocytic pool, which is also connected to the mobilization of SV to the active zone [45]. The periphery of the bouton showed a larger increase (or mobilization) of SVs in Atl-KD larvae than in control larvae (Fig. 2C–E). This increased density of the SV marker in response to stimulation may be due to enhanced recruitment of SVs to the bouton's periphery, but it may also be due to a decrease in release. As an alternative, changes to vesicle recycling might produce the same phenotype. On the other hand, disruptions in the actin cytoskeleton are a consequence of sustained activation of the BMP signal, which suggests that the high basal SV density observed in Tkv-CA larvae is the result of the disruption of the actin cytoskeleton involved in the transport of SV affecting the basal distribution and mobilization of SV [46].

The SV population has been divided into three functional populations or pools: A) the readily releasable pool, which primarily represents the SV population in the active zone; B) the recycling pool, which is mobilized to the active zone after the release of the previous pool; and C) the reserve pool, which corresponds to the SV population that is only released during intense stimulation. The exo-endocytic pool is an SV pool that is prepared to be released and recycled and is recruited in response to light to moderate synaptic stimuli. It has been proven that the distinct functional populations of SV differ in their availability for stimulus-dependent mobilization [47]. Despite the functional identification of these pools, it has not been possible to structurally separate these groups to date because SV mobilization causes population interchange and redistribution in the bouton’s space [47, 48]. This suggests that there is not one pool responsible for the differences in density distribution and SV mobilization observed in Atl-KD and Tkv-CA receptor overexpressed larvae. The release pool observed by the unload of FM 1–43 increased significantly in Atl-KD larvae but not in Tkv-CA larvae (Fig. 3) supporting that the SV mobilization deficiency is independent of BMP signaling. The exo-endocytic pool is the primary pool mobilized by the FM 1–43 discharge protocol, which is labeled by the load protocol without recruitment compensation or interchange between the other pools [49, 50]. The fact that changes in the density and mobilization of peripheral CSPs, where the active zones are located, are noticeable following synaptic stimulation may potentially be connected to this phenotype.

Regarding BMP signaling, the levels of loading and unloading of FM1-43, comparable to the control, in Tkv-CA larvae and *wit*/ + mutants, suggest that this phenotype is not sensitive to modifications in BMP signaling. The fall in the abundance of SV markers, the increase in their density and mobilization to peripheral zones of the bouton, after stimulation and the greater mobilization of the exo-endocytic population evidenced in the Atl-KD larvae, could be related to the reported inability to recover the synaptic function after a sustained tetanic stimulus, where the modifications in the intracellular traffic of SVs would be sufficient to respond in early phases of this stimulus, but they would not be able to maintain a sustained compensatory response over time [42]. Regarding Atl and SV, it is interesting that, in rat brain Atl was identified as one of the GTPases included in the SV proteome [51]. This, together with the results obtained here, suggests that Atl-KD modifies the synaptic activity-dependent mobilization of SV and that this protein play a regulatory role in the intracellular traffic of SV.

To sustain synaptic homeostasis and neurotransmission, endocytosis, recycling, and degradation activities must be properly balanced. Rab proteins Rab 5, Rab 4, Rab 11, and Rab 7 are engaged in the SV cycle [52–55]. In addition to the accumulation of SV and lysosomal markers in the distal axons, De Gregorio et al. [42] reported an increase in the formation of multivesicular body-like structures in the synaptic bouton, which raises the possibility that the organization and life cycle of the SV changes as a result of Atl-KD [56]. Here we revealed that Atl-KD modifies the accumulation of SV in the endosomal compartments positive for Rab 11, but not for Rab 4 (early recycling endosome) or Rab 7 (late endosome) (Fig. 4). This suggest that in motor neurons, the loss of Atl function selectively affects the traffic of SV through this late recycling endosome. Also, both Atl and Rab 11 have been identified as SV components [51], thus Atl could modify the destination or transit of the vesicles to or from this compartment. As a potential mediator of this process, we could name Protrudin, a resident protein of the ER that has been reported to associate with Atl, presents interaction domains with Rab 11 [57, 58] and is implicated in the symptoms of HSP. Rab 11 is a key presynaptic regulator during strong synaptic stimuli [59]. The synaptic phenotypes of increased density and peripheral mobilization of CSP described here are observed after synaptic stimulation, together with the enhanced discharge of the labeled exo-endocytic pool. Rab 11 phenotype, however, is evident in unstimulated conditions, suggesting that endosomes compartments underline the SV mobilization phenotypes. Additionally, this phenotype could be related to the multivesicular body-like structures previously observed in Atl-KD larvae, a compartment that also interacts with Rab11 and where this protein performs biogenesis, transport, and secretion functions [42, 60, 61].

Numerous neurological diseases, such as Huntington's disease and amyotrophic lateral sclerosis, which are linked to differences in BMP signaling as well as changes in synaptic function, have been also linked to modifications in the function of Rab 11 [62, 63]. Regarding BMP signaling, it has been described that mutations of Rab 11 or of the F-BAR / SH3 protein Nervous Wreck, which co-localizes with Rab 11 and exerts a role in the negative regulation of BMP by the recycling of its receptors, is associated with a loss of inhibition of this pathway and an increased development of the motor neuron, observed as an increase in the number of synaptic boutons [64, 65]. The lack of BMP inhibition in these larvae may be related to problems in the movement of activated receptors in this endosomal recycling compartment, according to the similarity of their morphometric phenotypes.

## Conclusions

Together, these findings point to a novel mechanism by which the loss of function of Atl, an axonal ER structuring protein in motor neurons, associates to defects in SV distribution and release independent of the increased signaling of the BMP pathway. This would imply that HSPs caused by *atlastin* mutations associate to synaptic function defects, and that these defects could be detected before the distal axonopathy characteristic of the disease.

## Methods

### Drosophila stocks

Several *Drosophila* strains were used, including: *w*^*1118*^ (Bloomington, USA, BDSC); *wit*^A12^ (BDSC); *C380-GAL4* (BDSC); *OK6-GAL4* (BDSC), UAS-*dicer2*; UAS-dsRNA-*atl* (Vienna Drosophila Resource Center, Austria, VDRC), UAS-*dicer2*, UAS-*tkv*-CA, UAS-rab4-GFP, UAS-rab11-GFP and Rab7-YFP (BDSC). A detailed table with all the stocks used can be found in Additional file 1: Table S1.

### Confocal microscopy

For morphometric analysis, pMAD and synaptic vesicle marker (CSP and VGLUT) fluorescence images were acquired with an Olympus FluoView 1000 confocal microscope, 60X objective add N.A. 1.35. and oil medium, 4X zoom and Z step 0.5 µm. Images for FM 1–43 analysis were acquired with an Olympus BX61WI Spinning Disc microscope, with a 60X objective add N.A. 1.42 and oil medium and 0.5 µm Z-step.

### STED microscopy

Images for CSP distribution and colocalization with rab proteins analysis were acquired using a Leica TCS Sp8 STED microscope with a 93X objective add N.A. 1.3 and glycerol medium, 5X zoom, reaching 27.5 nm resolution in XY and 0.1145 µm in Z. STED images were deconvoluted using the Huygens Scripting program (Scientific Volume Imaging, Hilversum, The Netherlands).

### Immunohistochemistry

For larvae body wall dissections and immunostaining protocols were performed as Duncan, Lytle, Zuniga, & Goldstein [66], respectively. We made some modifications to this protocol, including a shorter fixation time (20 min) and a stronger PBT solution (0.3% PBS plus triton X-1000). For STED analysis, the same protocol was used, including differences in the secondary antibodies’ concentrations used. For both confocal and STED microscopy, samples were mounted using the mounting medium Vectashield H-1000 (Vectorlab, USA). A detailed table with all the primary and secondary antibodies used can be found in Additional file 1: Table S2.

### Antibodies

Primary antibodies: α-DCSP (1:200; Developmental Studies Hybridoma Bank, USA; DHSB), α-pMAD (1:300, Millipore-Sigma), α-VGLUT (1:10,000, kindly gift from A. DiAntonio, Daniels 2004). Confocal secondary antibodies: Cy5-Hrp y Alexa 594-Hrp, Alexa 488-Hrp, Rhodamine TRITC, FITC and Cy5 α-Mouse or α-Rabbit (1:300, Jackson ImmunoResearch, USA). STED secondary antibodies: Abberior^®^Ster580 FluoTag-X4- α-GFP, Atto 647N α-Mouse, Atto 594 α-Rabbit (1:200 and 1:300, respectively, Nano-Tag Biotechnologies, Germany), Alexa 594-Hrp; Alexa 488-hrp (1:300, Jackson, USA).

### FM 1–43 assays

Larvae dissection was performed as described in Smith & Taylor [67]. FM 1–43 assay was performed using the protocol described by Gaffield and Betz [68]. Dissected larvae were incubated in 5 µM of FM 1–43 (Invitrogen, USA # T35356) for 3 min in 90 mM KCL in HL3.1 for neuronal stimulation. After stimulation, the larvae were washed 3 times with HL3.1 without calcium and 100 µm of ADVASEP-7 (Biotum, USA #70,029) to remove the dye from the surface. Then the larva is incubated in HL3.1 medium and 0.5 mM calcium when images of FM 1–43 loading are acquired. For FM 1–43 unloading, a second chemical stimulation is performed, in absence of FM 1–43 dye addition. Finally, the larva is washed and incubated in HL3.1 with EGTA for the FM 1–43 unloading images.

### Quantification and Statistical analysis

Synaptic bouton number, pMAD and synaptic vesicle markers intensity quantification, as well as FM 1–43 assays were determined in the larvae motor neurons present in the A6 abdominal segment, between muscles 6 and 7. Synaptic bouton quantification was performed using an HRP antibody allowing axonal membrane labeling, while synaptic bouton visualization was attained using α-CSP and α-dVGLUT antibodies. dVGLUT and dCSP synaptic vesicle labels, as well as pMAD label intensity was determined as the average fluorescence intensity of these markers in the synaptic bouton.

We selected the population of synaptic boutons that presented an area size between 2.5–12 µm^2^ to study (type Ib), with exception to the CSP density quartile analysis, in which we quantified boutons of all sizes. This range was determined by measuring the area (µm^2^) of all the synaptic boutons of 10 control larvae in the A6 abdominal segment (muscles 6/7), using α-HRP, to mark the motor neuron membrane and α-CSP for SV. From these data, a size distribution histogram was elaborated and subsequent curve fitting using a Bayesian information criterion (BIC).

Fluorescence intensity quantification in the synaptic bouton was performed as in Andlauer and Sigrist [69], with slight modifications due to the extended nature of these antibodies labeled inside the bouton (compared to the discrete structures analyzed by these authors). For each independent experiment (at least 2 for each experimental group and driver), all images were acquired using the same optic parameters. ImageJ software (NIH, U.S.A.) was used for image analysis. For each independent experiment, the average of all image backgrounds (Bk) was subtracted before antibody fluorescence quantification. For fluorescence quantification, using the HRP label we determined the maximal area as a ROI for each synaptic bouton. where synaptic vesicle fluorescence was quantified. These ROIs were used in the SV marker channel, defining the image representing the maximum area of the bouton as the center of the bouton. After obtaining the value of the average intensity of that optical section, the average intensity of the 2 images above and below this section was calculated and added, obtaining a representative sample of the signal contained in each bouton.

ImageJ was also used for FM 1–43 image analysis. For this, from the dye loading images we selected a ROI of each synaptic bouton, using the optical section that represented its maximum area, where we calculated the average fluorescence intensity in each ROI. The average intensity of the Bk of the non-synaptic region located immediately lateral to the bouton was also quantified, which, together with the remote Bk of the image, were averaged and subtracted in each final intensity calculation for each bouton. This calculation makes it possible to rule out that muscle Bk contributes to the final calculation of the average intensity of each bouton. With this method, the load average was calculated, including all quantized synaptic boutons. This same procedure was calculated for the FM 1–43 discharge, where the average fluorescence, calculated from each synaptic bouton present in the discharge image, was divided by the average load of the same larva. This final discharge /load ratio was subtracted from 1, obtaining the discharge percentage. Final values were normalized to control levels within each experiment.

### STED analysis

Program A: Developed in Matlab (Mathworks, USA) by Benedikt Auer, programmer from the LIN institute, with collaboration of Dr. Ulrich Thomas. The program generates a distribution map of the local maxima in the CSP mark, identifying the maximum signal values among neighboring pixels. Here, the CSP confocal, CSP STED and RAB STED channels of the image file were used. Briefly, a ROI for each synaptic bouton will be selected from the CSP confocal channel, this will be used as a mask of the respective bouton. Then a Z-stack and the borders of the bouton will be defined. Finally, the CSP STED and RAB STED channel will be selected and analyzed. For each synaptic bouton, two zones are analyzed: A) the peripheral zone, comprising the first 200 nm from the synaptic bouton’s outer edge and where the active zones are located, and B) the central zone, comprising the rest of the inner mark inside the bouton area (Fig. 2B) [70]. The program creates binary mask images of the boutons, quantifies the bouton areas and the number of local CSP maxima (hereinafter referred to as CSP particles) and density, and, also, creates a CSP particle distribution map. These were used as input for Program B (see below).

Program B: A macro for the Fiji software [71] written by Jorge Jara-Wilde (SCIAN-Lab, BNI/U. of Chile) as a post-processing step for the CSP distribution maps and bouton masks generated with Program A. The macro automates the measurement of CSP density variations from the bouton periphery inwards. Starting from the bouton binary image, the total area of the bouton and the CSP density within it are computed. Then, an iterative area shrinking of the bouton region towards its center is implemented by successive morphological erosions. At each iteration, the CSP density is measured within the eroded bouton area. Then, the density of CSP is quantified and accumulated together with the variation of the bouton area, encompassing its entire structure.

### Colocalization analysis

Colocalization analysis images were acquired at Leibniz-Institut für Neurobiologie (LIN, Magdeburg, Germany), using STED microscopy. For each analysis, the STED channel images for CSP and Rab marker were used, as well as the HRP confocal channel, which allowed delineation of the edge of each synaptic bouton. Using ImageJ, for each synaptic bouton we performed a HRP signal mask, located along the entire Z-axis of each bouton, which was used as ROI to select all the optical slices containing the CSP and Rab information included in that region. In order to determine the colocalization coefficients of both channels, using the JACoP plug-in [72], the CSP and Rab signal were segmented, and the Manders colocalization coefficients M1 and M2 were determined. Here, M1 corresponds to the amount of signal contained in colocalized pixels of the CSP channel divided by its total fluorescence, and M2 corresponds to the same ratio, but for the Rab channel. These coefficient values give an estimate of the percentage of colocalization of one signal over the other [72]. This colocalization analysis was performed for CSP with the endosomal markers Rab4, Rab7, and Rab11.

### Statistical analysis

Graphpad Prism 6 software (GraphPad Software, USA) was used for all statistical analysis. Prior to determining the statistical test to be used, a Kolmogorov–Smirnov (KS) normality test was performed. After determining the distribution of the samples analyzed, comparisons between groups were realized using Student’s t-test, Mann–Whitney (MW), parametric ANOVA or Kruskal–Wallis (KW) tests, followed by Tukey or Dunn's multiple comparisons, respectively. In case of two-way analysis, a two-way ANOVA was performed, followed by Tukey and Sidak post-hoc tests.

### Supplementary Information

**Additional file 1: Figure S1.** Atl-KD in motoneurons (OK6) increases synaptic pMAD and morphometric parameters. **Figure S2.** Atl-KD in motor neurons does not modify synaptic markers levels, in Rab´s overexpression background. **Figure S3.** Comparison of synaptic bouton number (S3A) and Satellite bouton number (S3B) between all genotypes using normalized data. **Figure S4.** Atl-KD in motoneurons modifies pMAD and CSP intensity, NMJ morphometric parameters and FM 1-43 unloading. **Figure S5.** CSP Intensity quantified by area of the bouton divided in quartiles. **Table S1.** *Drosophila* stocks. **Table S2.** *Drosophila* antibodies used for immunostainings.

## Data Availability

Data sharing is not applicable to this article as no datasets were generated or analysed during the current study.
