# Supplementary material for: Drosophila Atlastin regulates synaptic vesicle mobilization independent of bone morphogenetic protein signaling
Source: Biol Res. 2023 Sep 14;56:49. doi: 10.1186/s40659-023-00462-1 (PMC10503011; doi:10.1186/s40659-023-00462-1)
Supplement: Supplementary file 1 — Additional file 1: Figure S1. Atl-KD in motoneurons (OK6) increases synaptic pMAD and morphometric parameters. Figure S2. Atl-KD in motor neurons does not modify synaptic markers levels, in Rab´s overexpression background. Figure S3. Comparison of synaptic bouton number (S3A) and Satellite bouton number (S3B) between all genotypes using normalized data. Figure S4. Atl-KD in motoneurons modifies pMAD and CSP intensity, NMJ morphometric parameters and FM 1-43 unloading. Figure S5. CSP Intensity quantified by area of the bouton divided in quartiles. Table S1. Drosophila stocks. Table S2. Drosophila antibodies used for immunostainings. [file 40659_2023_462_MOESM1_ESM.docx]

**Additional file figures**

**
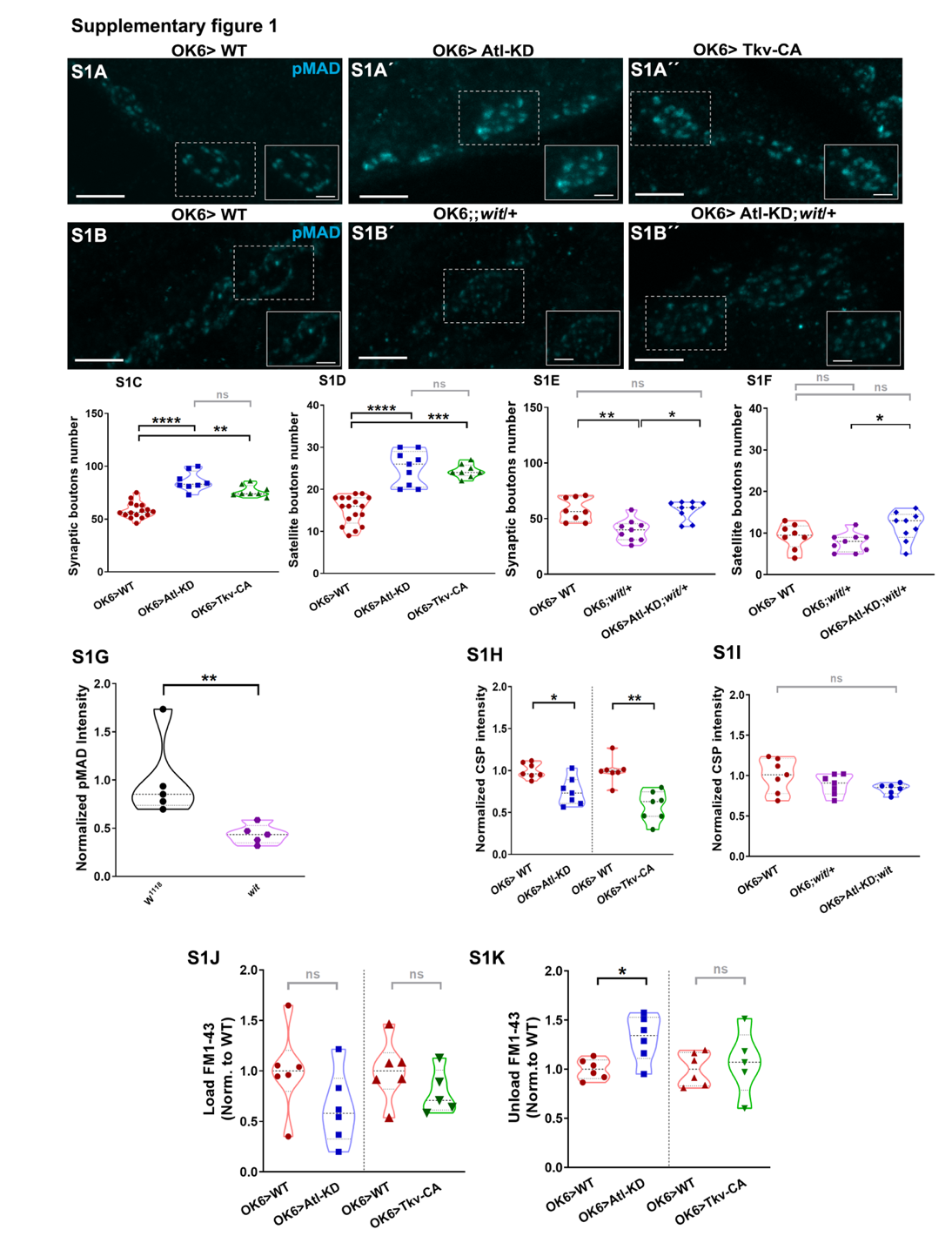
**

**Additional 1: Figure S1**: **Atl-KD in motoneurons (OK6) increases synaptic pMAD and morphometric parameters.** A, B) Representative confocal images (maximum intensity Z projection) of synaptic pMAD from control (OK6), Atl-KD, constitutive active TkvCA, *wit* mutant (*wit*) and Atl-KD; *wit* larvae. pMAD antibody stain is in cyan color. Scale bar of large image: 5µm, of cropped image: 2µm. C-F) Quantification of synaptic and satellite bouton number of the different genotypes. Each scatter dot represents one larva measurement. Kruskal-Wallis, p-value *<0,05; **<0.01; ****<0.0001; n=7. G) quantification of synaptic pMAD intensity from w1118 and *wit* mutant larvae. The *wit*/+ mutant reduces synaptic pMAD in the motor neuron. Mann-Whitney, p-value *<0,05; n=5 larvae. H-I) normalized synaptic CSP intensity of different genotypes.
J-K) Normalized fluorescence of FM1-43 for Load and Unload conditions of the different genotypes. Each scatter dot represents one measurement.

Mann-Whitney, p-value *<0,05; n=7.


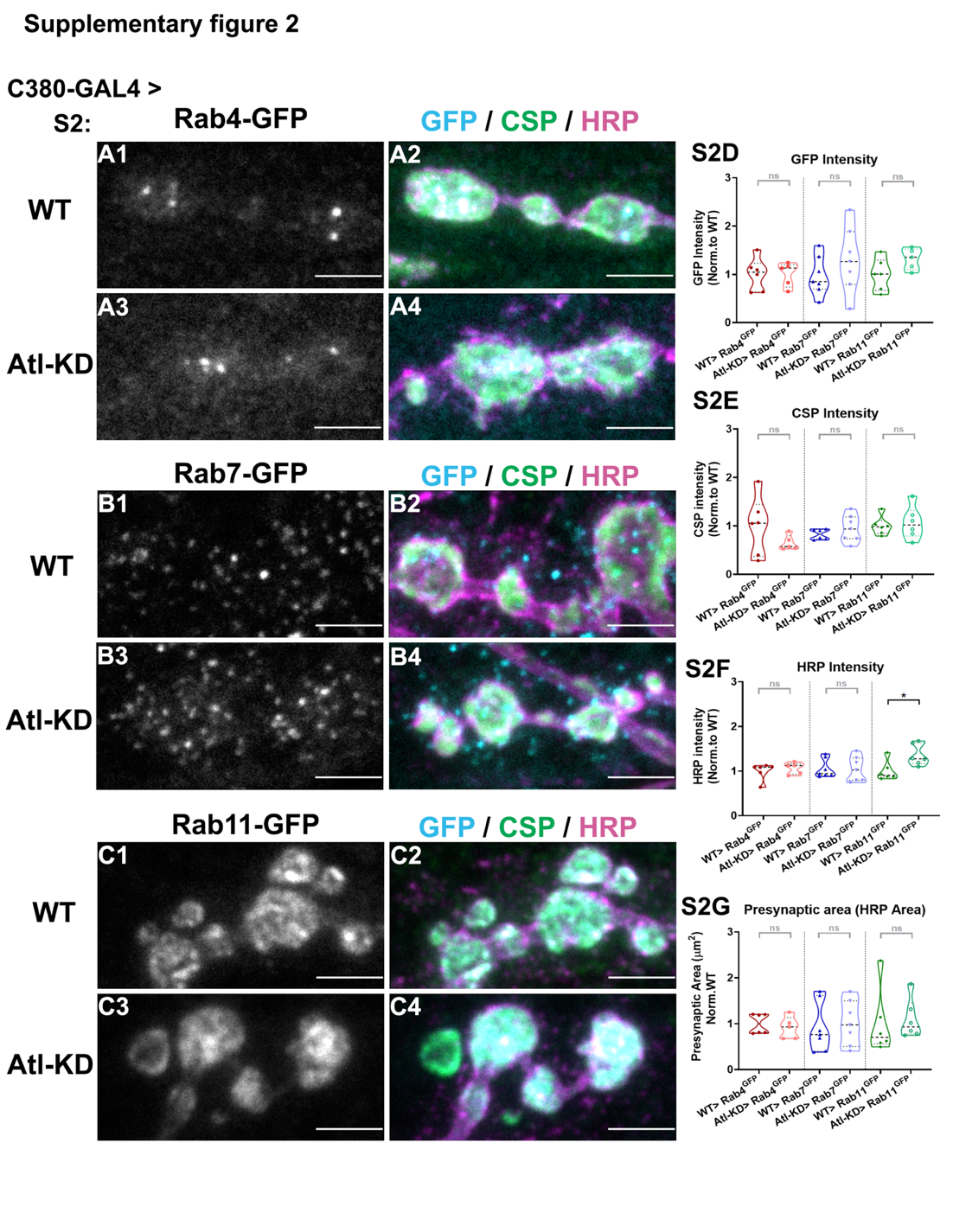


**Additional 1: Figure S2**: **Atl-KD in motor neurons does not modify synaptic markers levels, in Rab´s overexpression background.** A-C) Representative confocal images (maximum intensity Z projection) of synaptic markers of (C380-Gal4) control and Atl-KD, overexpressing Rab4-GFP (A1-4), Rab7-GFP (B1-4) and Rab11-GFP (C1-4). GFP staining is labeled in gray color (A1,3; B1,3; C1,3) and cyan (A2,4; B2.4; C2,4), CSP in green and HRP in magenta (A2,4; B2.4; C2,4). Scale bar: 2µm. D-F) Quantification of fluorescence intensity of synaptic GFP (D), CSP (E) and HRP (F) intensity of control and Atl-KD larvae, overexpressing Rab (4,7,11)-GFP. Mann-Whitney, p-value *<0,05; n=6 larvae. G) Presynaptic area, delimited by HRP staining of control (C380) and Atl-KD larvae, overexpressing Rab (4,7,11)-GFP. Mann-Whitney; n=6 larvae.


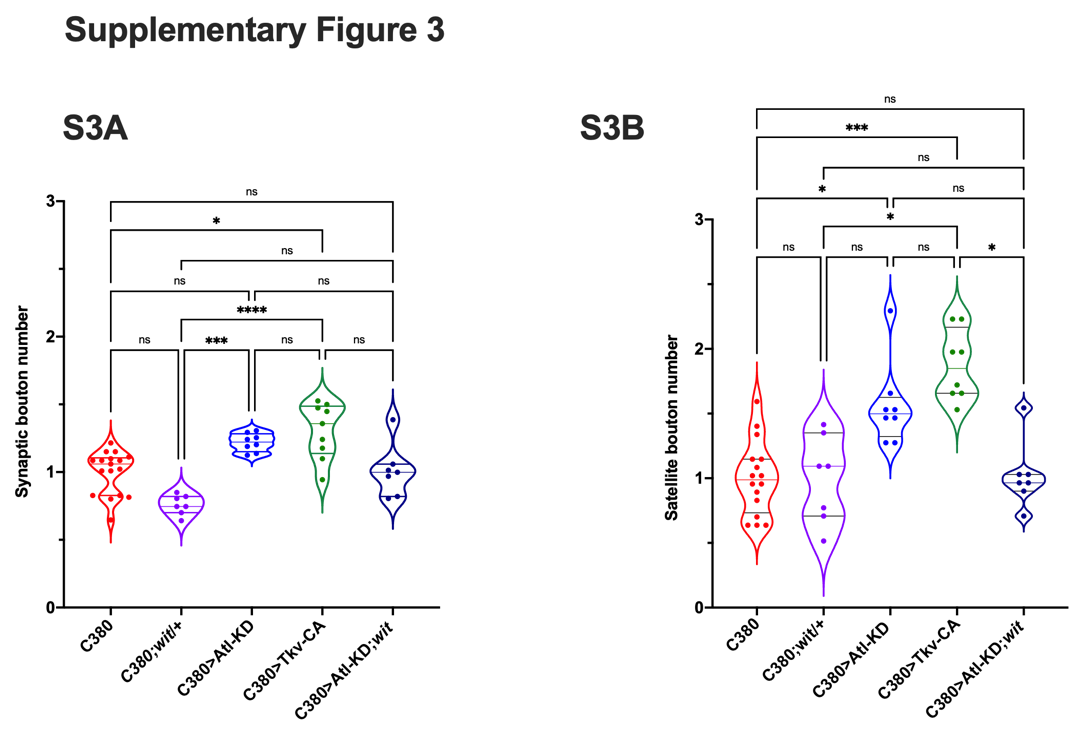


**Additional 1: Figure S3:** Comparison of synaptic bouton number (S3A) and Satellite bouton number (S3B) between all genotypes using normalized data.


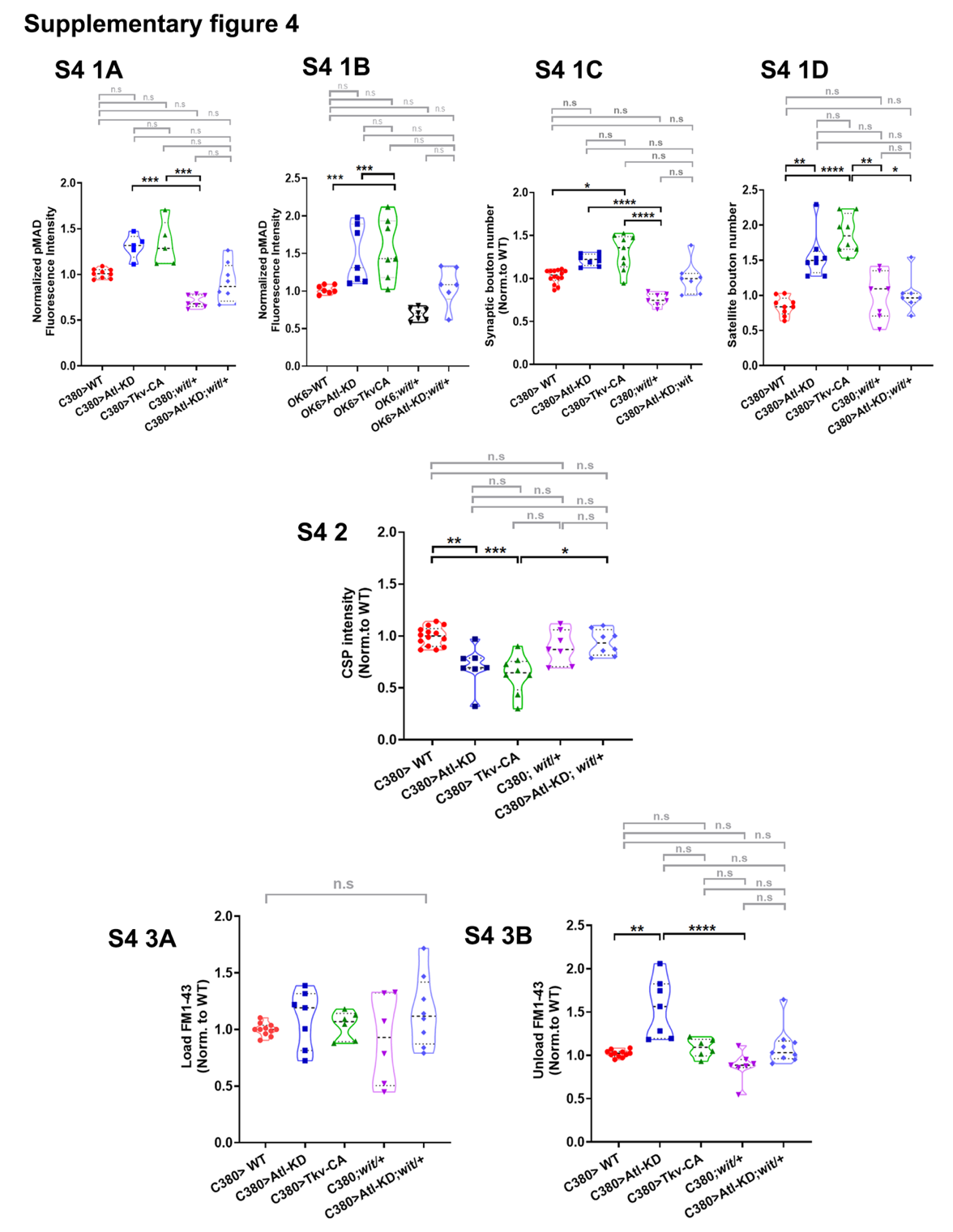
**Additional 1 Figure S4**: **Atl-KD in motoneurons modifies pMAD and CSP intensity, NMJ morphometric parameters and FM 1-43 unloading.** S4-1A-B) Normalized pMAD intensity quantification of larvae expressing Atl-KD, Tkv-CA and *wit* and Atl-KD; *wit* larvae using the motoneuron drivers C380 (S41A) and OK6 (S41B), pMAD intensity was normalized to the pMAD intensity levels of the control (C380 or OK6). Kruskal-Wallis, p-value *<0,05; **<0.01; ***<0.001. n=7-9 larvae. S4-1C-D) Synaptic and satellite bouton number of control (C380), Atl-KD, Tkv-CA, *wit,* and Atl-KD; *wit* larvae. Each scatter dot represents one measurement. Kruskal-Wallis, p-value *<0,05; **<0.01; ***<0.001; ****<0.0001. n=7-9 larvae. S4-2) Normalized CSP intensity quantification in larvae with Atl-KD, Tkv-CA and *wit* and Atl-KD; *wit* larvae using the motoneuron C380-Gal4 , CSP intensity was normalized to the CSP intensity levels of the control (C380). Kruskal-Wallis, p-value *<0,05; **<0.01; ***<0.001. n=7-9 larvae. S4-3A-B) Quantification of the fluorescence intensity of FM 1-43 normalized to control levels after the loading (3A) and unloading protocol (3B), of control (C380), Atl-KD, Tkv-CA, *wit* and Atl-KD; *wit* larvae. Each data point represents the average of several boutons in one larva. Kruskal-Wallis; p-value: **<0.01; ***<0.001; n=7-9 larvae.

**
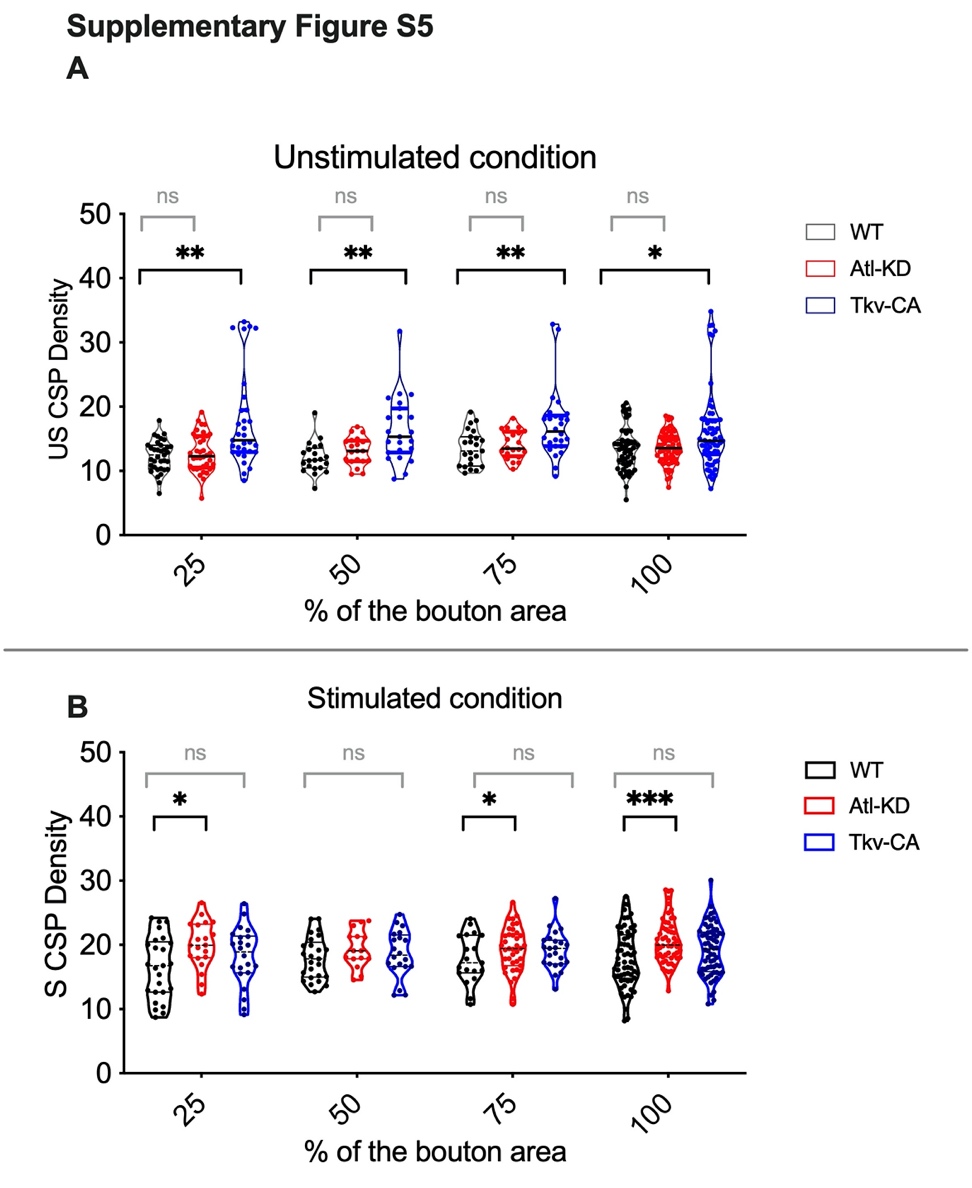
Additional file Figure S5**:**CSP Intensity quantified by area of the bouton divided in quartiles.** A) Comparison between genotypes of the CSP intensity in resting condition (unstimulated) in four areas from the more central area (25 %) to the more peripheric area (100 %) B) Comparison between genotypes of the CSP intensity after stimulation with potassium (stimulated) in four areas from the more central area (25 %) to the more peripheric area (100 %).

**Supplementary materials**

Table 1: *Drosophila* stocks

| **Genotype** | **Description** | **Origin** | **Reference** |
| --- | --- | --- | --- |
| *w1118* | wild-type strain | Not determined | De Gregorio et al. 2017 |
| *C380-GAL4* | GAL4 line for motor and sensory neurons | Gentil gift from V. Budnik, UMASS | Sanyal, 2009 |
| *Ok6-GAL4* | GAL4 line for motor neurons | Bloomington #64199 | Sanyal, 2009 |
| *UAS-dsRNA-atl* | double stranded (ds) RNA used for Altastin knockdown | VDRC #6719 | Klemm et al., 2013; Rao et al., 2016; Summerville et al., 2016; De Gregorio et al., 2017 |
| *wit* | Null mutant of Wit receptor | Bloomington #5173 | Aberle et al., 2002; Rodal, Motola-Barnes, and Littleton 2008; Smith et al., 2012; M. J. Sulkowski et al., 2016; Kim et al., 2019 |
| *UAS-tkv-CA* | UAS line used for constitutively active Tkv protein expression. The original strain was then balanced over TM6B,Tb | Bloomington #36536 | Khuong et al., 2010; M. Sulkowski, Kim, and Serpe 2014 |
| *UAS-rab4-GFP* | UAS line used for Rab4-YFP expression | Bloomington #9767 | (Nagel et al., 2017; Jiang et al., 2019) |
| *UAS-rab11-GFP* | UAS line used for Rab11-GFP expression | Bloomington #8506 | Steinert et al. 2012; Martin-Peña and Ferrus 2020 |
| *rab7-YFP* | Expresses endogenous Rab7-EYFP protein | Bloomington #62545 | Dunst et al. 2015; S. Y. Wang, Zhao, and Rodal 2019 |
| *UAS-dicer* | UAS line used for dicer expression. | Bloomington #24651 | Dietzl et al. 2007; Chen and Ganetzky 2012; Yamanaka et al. 2013 |
| *MKRS/TM6b* | Strain balanced on the III chromosome | Not determined | De Gregorio et al., 2017 |
| *if/CyO; MKRS/TM6b* | Strain double balanced on the II y III chromosome | Not determined | De Gregorio et al., 2017 |

Table 2: *Drosophila* antibodies used for immunostainings

| **Antibodies** | | | |
| --- | --- | --- | --- |
| **Antibody** | **Description** | **Host** | **Origin** |
| **α-CSP** | Antibody against Drosophila CSP | mouse | DSHB, EEUU #AB-528183 |
| **α-VGLUT** | Antibody against Drosophila CSP VGLUT | Rabbit | gentil gift from Dr. Di Antonio |
| **α-pMAD** | Antibody against phosphorylated MAD | Rabbit | Millipore #ABE2871 |
| **Cy5-HRP** | Antibody against phosphorylated HRP | Goat | Jackson ImmunoResearch, EEUU |
| **Alexa 594-HRP** | Antibody against phosphorylated HRP | Goat | Jackson ImmunoResearch, EEUU |
| **Alexa 488-HRP** | Antibody against phosphorylated HRP | Goat | Jackson ImmunoResearch, EEUU |
| **Rhodamine TRITC** | Antibody against mouse | Mouse | Jackson ImmunoResearch, EEUU |
| **FITC** | Antibody against mouse | Mouse | Jackson ImmunoResearch, EEUU |
| **FITC** | Antibody against rabbit | Rabbit | Jackson ImmunoResearch, EEUU |
| **Cy5** | Antibody against mouse | Mouse | Jackson ImmunoResearch, EEUU |
| **Cy5** | Antibody against rabbit | Rabbit | Jackson ImmunoResearch, EEUU |
| **Abberior®Star580** | Antibody against GFP |  | Nano-Tag Biotechnologies, Germany #NAT-N0304-AB580 |
| **Atto 647N** | Antibody against mouse | Mouse | Nano-Tag Biotechnologies, Germany |
| **Atto 594** | Antibody against rabbit | Rabbit | Nano-Tag Biotechnologies, Germany |
